# Isolation and identification of a new strain of hirame rhabdovirus (HIRRV) from Japanese flounder *Paralichthys olivaceus* in China

**DOI:** 10.1186/s12985-017-0742-4

**Published:** 2017-04-07

**Authors:** Jialin Zhang, Xiaoqian Tang, Xiuzhen Sheng, Jing Xing, Wenbin Zhan

**Affiliations:** 1 Laboratory of Pathology and Immunology of Aquatic Animals, Ocean University of China, No.5 Yushan Road, Qingdao, 266003 China; 2 Laboratory for Marine Fisheries Science and Food Production Processes, Qingdao National Laboratory for Marine Science and Technology, No.1 Wenhai Road, Qingdao, 266071 China

**Keywords:** Hirame rhabdovirus, *Paralichthys olivaceus*, Identification, Pathogenicity

## Abstract

**Background:**

Hirame rhabdovirus virus (HIRRV) is a rhabdovirus that causes acute hemorrhage disease in fish culture, resulting in a great economic loss in parts of Asia and Europe.

**Methods:**

In this study, we isolated a virus strain named as CNPo2015 from cultured Japanese flounder in Shandong province, China. Cell isolation, electron microscopic observation, RT-PCR detection and phylogenetic analysis were used for virus identification. Further, artificial infection experiment was conducted for virulence testing.

**Results:**

The gross signs included abdominal distension, fin reddening and yellow ascitic fluid in the abdominal cavity. Histopathological examination revealed marked cell degeneration and necrosis in the kidney. The tissue homogenates induced obvious cytopathic effects in EPC, FHM and FG cell lines. Electron microscopic observation showed the virus had a bullet-like shape with a capsule membrane. RT-PCR and sequencing analysis revealed that CNPo2015 belonged to the HIRRV with high sequence identity to HIRRV isolates. Infection experiment confirmed that the HIRRV CNPo2015 strain was virulent to flounder juveniles with a LD_50_ value of 1.0 × 10^5.9^ TCID_50_/fish.

**Conclusion:**

In conclusion, we described the first isolation and characterization of a HIRRV from Japanese flounder in China. This will provide a candidate material for further research on the infection mechanism and preventive strategies of HIRRV.

## Background

Hirame rhabdovirus (HIRRV) is a single-stranded RNA virus, which belongs to the genus *Novirhabdovirus* within the family *Rhabdoviridae*. The virus was first described in cultured Japanese flounder in Japan in the early 1980s [1], from where it gradually spread to South Korea and China [2, 3]. However, due to increasing travels and rapid globalization, the outbreak of HIRRV has also been reported in part of Europe [4]. HIRRV can affect a wide range of marine fishes including Japanese flounder, stone flounder (*Kareius bicoloratus*), black seabream (*Acanthopagrus schlegeli*) and sea bass (*Lateolabrax maculatus*) [5]. The major clinical signs of HIRRV infection are congestion of the gonads, focal haemorrhage of the skeletal muscle and fins and accumulation of ascitic fluid [1]. Nowadays, the lack of vaccines and drugs against HIRRV highlights the urgency and significance of investigating infection mechanism and preventive strategies against HIRRV [6].

As with all novirhabdoviruses, the HIRRV genome encodes six viral proteins including nucleoprotein (N), phosphoprotein (P), matrix protein (M), glycoprotein (G), non-structural (NV) and RNA polymerase protein (L) [7]. Among them, the G gene is relatively well conserved and often used as the target for HIRRV detection [8]. Sun et al. has established a reverse transcription PCR based on the G gene, which could specifically detect the HIRRV from other viruses [9]. The G protein also contains antigenic determinants that can induce antibodies in fish [10]. The DNA vaccine containing G gene could induce protective immunity against HIRRV infection [11]. In addition, the P gene was usually employed for the phylogenetic and epidemiological studies [12]. As aforementioned, we chose the P gene as the target for phylogenetic analysis.

In the spring of 2015, a hemorrhage disease was observed in farmed Japanese flounder in Shandong province, China. Typical clinical signs exhibited by the diseased fish were congestion of the fins and accumulation of ascitic fluid. In the present study, we described the histopathological examination, cell culture isolation, electron microscopy, molecular confirmation and phylogenetic analysis. We further performed the artificial infection experiment to determine the virulence of the HIRRV CNPo2015 isolate.

## Methods

### Sample collection

In this study, diseased flounder juveniles (average weight of 30 g, average body length of 15 cm) come from a fish farm in Shandong province, China. The fish suffering from a hemorrhage disease were kept at a temperature ranged from 8 to 10 °C, and the cumulative mortality rate was approximately 20% in a month. Five diseased fish and five healthy fish were taken for parasitological, bacteriological and virological examinations. The gills and mucus scrapings from the skin were inspected for the presence of parasites. The kidneys and spleens were homogenized and subjected to bacteria culture on brain–heart infusion agar plates at 15 °C for 7 days. Histopathological examinations were carried out using the tissue sections.

### Virus isolation

The kidney, spleen, brain and gill tissues from five diseased fish were individually homogenized at a ratio of 1:10 (w/v) in M199 medium supplemented with 10% FBS, and 1% Penicillin-Streptomycin (Gibco). The tissue homogenates were filtered through a 0.22 μm filter membrane and were inoculated on four fish cell lines including EPC, FHM, FG and CAR cell lines, cultured in M199 medium supplemented with 4% FBS and 1% Penicillin-Streptomycin at 15 °C. The inoculated cell cultures were incubated at 15 °C for 7 days and examined daily for the presence of CPE. The cultures were centrifuged at l,500 × g at 4 °C for 20 min and the supernatants were immediately stored at −80 °C. The obtained virus was named CNPo2015.

### Virus titering

Three fish cell lines (EPC, FHM and FG) were used for virus titering. Prior to infection, the cells were transferred into 96-well plates and grew to monolayer cells. Serial 10-fold dilutions of the tissue homogenate were inoculated on three fish cell lines. The cultures were maintained in M199 medium supplemented with 4% FBS and 1% Penicillin-Streptomycin at 15 °C. The virus titers on the cell lines were determined by 50% endpoint dilution assays (TCID_50_) according to the method described by Reed and Muench [13].

### Electron microscopy

EPC cell monolayers were incubated with the virus culture at a MOI of 0.1 for 2 days. Then the cultures were collected and fixed in 2.5% glutaraldehyde in phosphate buffer overnight. Ultrathin sections were prepared as previously described [14]. The sections were placed on grids and examined under a Jeol JEM-1200 EX electron microscope.

### RT-PCR and sequencing

500 μL of culture supernatants was used for RNA extraction using TRIzol (Takara). The quality and quantity of RNA were checked using a NanoDrop ND-8000 spectrophotometer (Thermo Scientific). cDNA was synthesized from 1 μg of RNA using M-MLV kit (Takara), according to the manufacturer’s instructions. The resulting cDNA was used for PCR analyses of HIRRV, VHSV, IHNV, VNNV. The PCR was performed in a total volume of 25 μL containing 1 μL of cDNA, 2.5 U of Taq DNA polymerase (Takara), 5 μL of PCR reaction buffer, 0.5 μL of each primer (10 pmol) and RNase-free water. Normal EPC cell sample was used as the negative control. All the PCR amplifications were performed according to previous studies [9, 15–18], and the primer sets and PCR conditions were shown in Table 1. The products were examined by electrophoresis using a 1.0% agarose gel. Then the products were sequenced using the BigDye® v3.1 dye terminator and were analyzed on an ABI PRISM 3730 XL DNA Analyzer (Applied Biosystems, USA), following the manufacturer instruction. The obtained sequences were submitted in the NCBI website and blasted for similar sequences in the GenBank database.

**Table 1:** Primers for specific detection of five different viruses

Virus	Primers sequence	PCR condition^a^	Source
HIRRV	F: 5′-GTGCCAATGGTACACGGACAA-3′	55 °C/35	[9]
	R: 5′-TGATCTCCGCATGTGCCTCTA-3′		
VHSV	F:5′- ATGGAAGGAGGAATTCGTGAAGCG-3′	55 °C/35	[15]
	R:5′-GCGGTGAAGTGCTGCAGTTCC-3′		
IHNV	F: 5′-AGAGATCCCTACACCAGAGAC-3′	50 °C/30	[16]
	R: 5′-GGTGGTGTTGTTTCCGTGCAA-3		
VNNV	F: 5′-CGTGTCAGTCATGTGTCGCT-3′	55 °C/25	[17]
	R: 5′-CGAGTCAACACGGGTGAAGA-3′		
LCDV	F: 5′-GCTGCTGATTTCGAATATGG-3′	50 °C/30	[18]
	R: 5′-GCTTGCATAGGCTTCTTC-3′		

^a^PCR condition: annealing temperature/cycles

### Phylogenetic analysis

The ORF of the P gene was amplified for phylogenetic analysis. A pair of specific PCR primers (P-F: 5′-ATGTCTGATAACGAAGGAG-3′ and P-R: 5′-CTACCTCATGGTCTTCTTG-3′) were designed using Primer premier 5.0. The amplification was performed as follows: 94 °C for 5 min, followed by 30 cycles of denaturing at 94 °C for 1 min, annealing at 53 °C for 1 min, extension at 72 °C for 1 min and finally incubation at 72 °C for 10 min. The obtained PCR products were sequenced as described above, and the sequence was submitted to the GenBank database. The P gene sequence of CNPo2015 was compared with five other HIRRV isolates in GenBank. The sequences were aligned using Clustal software (version 1.81). Phylogenetic tree based on the P gene was constructed by MCMC method using the BEAST software (version 2.4.5) as previously described [19], and the phylogenetic tree was visualized using FigTree (version 1.4.3).

### Experimental infection

Healthy flounder juveniles (10 ~ 15 cm, about 30 g) were obtained from a farm in Rizhao, Shandong, China. Prior to the experiment, PCR assay was performed to confirm the fish free of HIRRV. All fish were held in seawater at a temperature of 10 °C. After acclimation for 7 days, a total of 150 fish were randomly divided into five groups (30 fish per group). Fish in each group were injected intraperitoneally with 100 μL of virus culture (10^7.5^, 10^6.5^, 10^5.5^, 10^4.5^TCID_50_ per fish). In addition, fish in the control group were injected with equivalent amount of M199 media. All fish were checked daily for clinical signs and mortalities for 14 days.

## Results

### Gross examination and histopathology

**Fig. 1:**
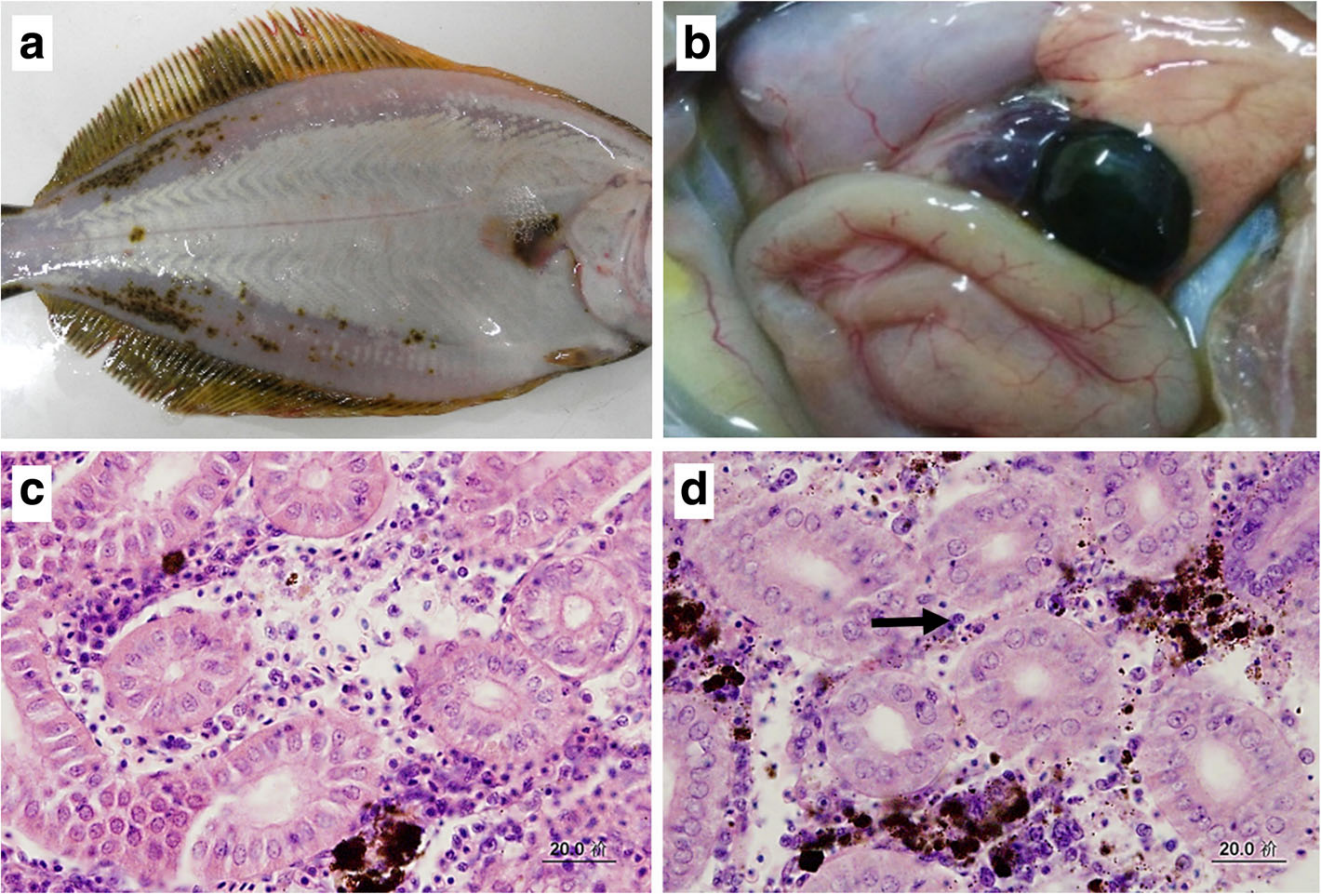
Clinical signs and pathologic changes of diseased *Paralichthys olivaceus*. **a** Symptoms on the surface of diseased fish showed congested fins, expanded abdomen and dark body color. **b** Gross appearance of visceral of diseased fish displayed signs including dark red spleen, congested intestine, and yellow ascitic fluid. **c** Kidney section of healthy fish. **d** Kidney section of diseased fish displayed pathologic changes including cell degeneration and necrosis (*arrow*), karyopyknosis with pieces of broken nucleus. Scale bars = 20 μm

The external clinical signs observed in the diseased fish were skin darkening, abdominal distension, fin reddening (Fig. 1a). After dissection, diseased fish showed visceral pallor, capillaries expansion and yellow ascitic fluid in the abdominal cavity (Fig. 1b). No bacteria or parasites were isolated from the fish samples. Histological examination showed that most of the kidney cells were characterized by marked cell degeneration and necrosis. The formation of broken nucleus and pathologic pigmentation were also present in kidneys (Fig. 1d).

### Virus isolation and titration

**Fig. 2:**
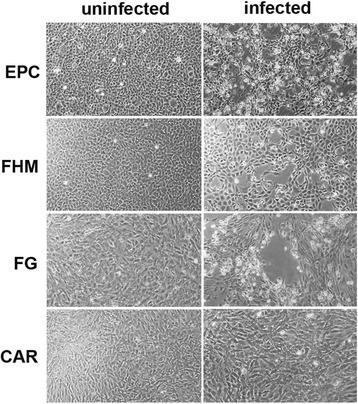
Virus isolation on four fish cell lines. Compared with uninfected fish cell lines, the infected EPC cell line showed typical CPE with cell rounding, detachment and dead cells. The infected FHM and FG cell lines were also observed with CPE. No CPE were observed in CAR cell line. Magnification, 30 ×

The homogenate supernatants of kidney and spleen tissues induced positive CPE in the EPC, FHM, FG cells at 3 days post inoculation at 15 °C. The CPE was characterized by rounded and granular cells, grape-like clusters, cell detached and lysis. No CPE was observed in the CAR cell line (Fig. 2). In all cell lines, no CPE was induced by the brain and gill samples. At 7 days post inoculation, the EPC cells produced the highest titer of virus with a titer of 1 × 10^8.5^ TCID_50_/mL, while the titer was 1 × 10^8.1^ TCID_50_/mL for FHM cells, and 1 × 10^7.3^ TCID_50_/mL for FG cells, respectively. Virus titers in all cell lines stabilized for the remainder of the infection.

### Electron microscopic observation

**Fig. 3:**
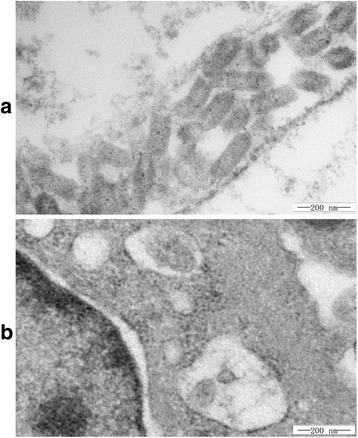
Morphology of the virions under electron microscope. **a** A large amount of virions aggregated on the surface of the cell. **b** Virions were wrapped by vesicles in the cytoplasm. Scale bars = 200 nm

Transmission electron microscopy showed that abundant viral particles were aggregated on the surface of cells at 2 days post inoculation (Fig. 3a). Meanwhile, some particles were also found inside the cytoplasm with a vesicle encapsulated (Fig. 3b). The intact virion exhibited a bullet-shaped capsid enclosed with envelope. Moreover, the virion averaged approximately 160 nm in length and 80 nm in width.

### RT-PCR detection

**Fig. 4:**
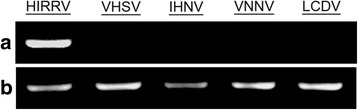
RT-PCR analysis. **a** RT-PCR amplification from infected EPC cells using specific primers of HIRRV, VHSV, IHNV, VNNV and LCDV. **b** The flounder β-actin was used as an internal control gene

As shown in Fig. 4, a specific band was amplified from virus culture supernatants using HIRRV specific primers, while no product was detected in the normal EPC cell samples (data not shown). No product was detected using primers of VHSV, IHNV, VNNV or LCDV. The G gene fragment of CNPo2015 revealed 100% sequence identity with the known HIRRV isolates.

### Phylogenetic analysis of the P gene

**Fig. 5:**
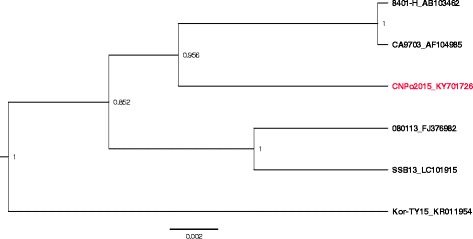
Phylogenetic analysis performed using P genes from six HIRRV isolates. The P gene sequence of the HIRRV CNPo2015 is in *red* color. The scale bar at the *bottom* indicates 0.002 nucleotide substitutions per site

The P gene of CNPo2015 strain was amplified and the complete ORF sequence was submitted to GenBank under accession number KY701726. The P gene sequence analysis showed CNPo2015 had the highest nucleotide sequence identity to 080113 strain and SSB13 strain (98.5%), followed by CA-9703 strain and 8401-H strain (98.1%) and Kor-TY15 strain (97.4%). A phylogenetic tree based on the P gene ORF sequences revealed that CNPo2015 strain was clustered in a clade with 8401-H strain and CA-9703 strain. It showed that CNPo2015 strain was farther to SSB13 strain, 080113 strain and Kor-TY15 strain, which were isolated from spotted sea bass, stone flounder and black seabream, respectively (Fig. 5).

### Experimental infection

**Fig. 6:**
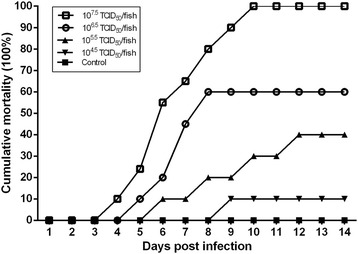
Cumulative mortality rates of flounder juveniles after challenge with different doses of the HIRRV CNPo2015 through day 14 post-infection. Each group contained 30 fish

Flounder juveniles were inoculated with different doses of the HIRRV CNPo2015 strain. Symptoms such as abdominal distension and fin reddening appeared on day 4 and began to die on day 5 post-infection. At 14 days post-infection, fish infected with 10^7.5^, 10^6.5^, 10^5.5^, 10^4.5^ TCID_50_ of CNPo2015 strain had cumulative mortalities of 100, 60, 40 and 10%, respectively (Fig. 6). Mortality rates in each group were plotted and the LD_50_ in juvenile flounder was determined to be 1.0 × 10^5.9^ TCID_50_/fish. No signs or mortalities were observed in the control fish during the experiment.

## Discussion

In China, the first HIRRV isolation was reported in cultured stone flounder in 2010 [3]. Although there were some cases discovered with similar symptoms in other marine fish, no formal reports on the virus identification have been reported. In 2015, a virus strain CNPo2015 was isolated from diseased Japanese flounder in Shandong Province, China. It caused severe symptoms such as visceral congestion and ascitic fluid in diseased fish, which were in accordance with the HIRRV infection cases [20]. Therefore, this study described a comprehensive understanding of the pathology of CNPo2015 with virus isolation, electron microscopy analysis, molecular comparison and virulence analysis. The results suggested that the CNPo2015 strain belonged to HIRRV.

HIRRV infection could cause severe disease with high morbidity and mortality in susceptible fish [21]. However, there are some factors that can influence the pathogenicity of rhabdovirus. It has been reported that water temperature plays important roles in the onsets and development of diseases [22]. High mortality of HIRRV-infected fish often occurred when water temperatures decreased under 15 °C, while the symptoms relieved when the water temperature rose above 15 °C [23]. In our study, a high mortality rate in juvenile flounders occurred when the temperature was about 10 °C. Additionally, the host age is also an important influence factor. Previous studies showed that adult flounders (100 ~ 250 g) and fry flounders (about 10 g) injected with 10^6^ TCID_50_ of HIRRV showed cumulative mortalities of 25 and 100% [1, 24]. In our study, we calculated a cumulative mortality of 60% in juvenile flounders (about 30 g) injected with the same dose of HIRRV. Therefore, age and water temperature can be considered the important factors for making preventive strategies of HIRRV.

Phylogenetic analysis confirmed that the P gene sequence of the CNPo2015 strain was similar with other HIRRV isolates (more than 97% sequence identity), which was consistent with a previous report [25]. Among the different HIRRV isolates, the CNPo2015 strain was more closely related to the HIRRV strains 8401-H and CA-9703, which were previously isolated from Japanese flounder in Japan and Korea, respectively. This is probably as a result of increasing global trade and introduction breeding that accelerate the virus transmission. Nevertheless, it was noted that all the known isolates of HIRRV shared a high sequence identity, which could be speculated that all the isolates may originate from a same population. Therefore, a complete genome sequence analysis might be needed for further assessment of the genetic relationship among the different HIRRV isolates.

## Conclusions

In conclusion, we described the isolation and characterization of a HIRRV isolate from Japanese flounder in China. The present isolate was closely related to known HIRRV isolates. It could infect diverse fish cell lines and induce obvious CPE. Result from the infection experiment revealed that CNPo2015 strain was virulent to juvenile flounder. Further studies will be focused on the infection mechanism and preventive strategies of HIRRV.

## Abbreviations

CAR: Carp fin
CPE: Cytopathic effects
EPC: Epithelioma papulosum cyprini
FBS: Fetal bovine serum
FG: Flounder gill
FHM: Fathead minnow
IHNV: Infectious hematopoietic necrosis virus
LCDV: Lymphocystis disease virus
LD_50_: Median lethal dose
MCMC: Markov chain Monte Carlo
MOI: Multiplicity of infection
ORF: Open reading frame
SVCV: Spring viremia of carp virus
TCID_50_: 50% tissue culture infective dose
VHSV: Viral hemorrhagic septicemia virus
VNNV: Viral nervous necrosis virus
